# Early escitalopram administration as a preemptive treatment strategy against spasticity after contusive spinal cord injury in rats

**DOI:** 10.1038/s41598-021-85961-5

**Published:** 2021-03-29

**Authors:** Youngjae Ryu, Toru Ogata, Motoshi Nagao, Yasuhiro Sawada, Ryohei Nishimura, Naoki Fujita

**Affiliations:** 1 Department of Veterinary Surgery, Graduate School of Agricultural and Life Sciences, The University of Tokyo, Tokyo, 113-8657 Japan; 2 Department of Rehabilitation for the Movement Functions, National Rehabilitation Center for Persons with Disabilities, Saitama, 359-8555 Japan; 3 Molecular Regeneration and Neuroimaging Laboratory, Burke Neurological Institute, Weill Cornell Medicine, White Plains, NY 10605 USA; 4 Department of Rehabilitation Medicine, The University of Tokyo Hospital, Tokyo, 113-8655 Japan

**Keywords:** Spinal cord diseases, Regeneration and repair in the nervous system, Spinal cord injury

## Abstract

In the majority of spinal cord injury (SCI) patients, spasticity develops in the subacute phase and chronically persists with muscle hypertonia. Among various pathological conditions underlying spasticity, upregulated expression of 5-HT receptors (5-HTR) on the spinal motor neurons due to 5-HT denervation is considered one of crucial factors for hyperexcitability of the spinal circuit. As a 5-HT signal modulator, selective serotonin re-uptake inhibitors (SSRIs) are ordinarily prescribed for diseases associated with 5-HT in the CNS, and are known for their ability to increase 5-HT levels as well as to desensitize 5-HTR. Here, we hypothesized that early SSRI administration as a preemptive treatment strategy would effectively prevent the onset of spasticity. We used a rat model of contusive SCI and administered escitalopram during the first 4 weeks after injury, which is the period required for spasticity development in rodent models. We performed a swimming test to quantify spastic behaviors and conducted the Hoffman reflex test as well as histological analyses for 5-HT_2A_R and KCC2 expressions. Four weeks of escitalopram administration suppressed spastic behaviors during the swimming test and reduced the population of spasticity-strong rats. Moreover, the treatment resulted in decreased immunoreactivity of 5-HT_2A_R in the spinal motor neurons. Result of the H-reflex test and membrane expression of KCC2 were not significantly altered. In summary, early escitalopram administration could prevent the onset of spastic behaviors via regulation of 5-HT system after SCI, but could not modulate exaggerated spinal reflex. Our results suggest a novel application of SSRIs for preventative treatment of spasticity.

## Introduction

Spasticity is defined as involuntary increased muscle tone and is a common symptom after upper motor neuron lesions, such as spinal cord injury (SCI)^1,2^. Clinically, 60% to 80% of SCI patients manifest spastic symptoms as muscle spasm, hypertonia, hyperreflexia, and clonus^2,3^. These symptoms are first absent, and they gradually emerge in the subacute phase after the injury and interfere with voluntary physical performances throughout the chronic phase. In human patients, characteristic spastic symptoms emerge around 2 months after SCI^4^. In spinalized rodent models, similar signs are observed, with progressive muscular stiffness and tail spasm starting 3–4 weeks after SCI^5,6^. To alleviate exaggerated muscle hyperactivity, a series of pharmacological drugs, such as gamma-aminobutyric acid (GABA) agonists, have been prescribed clinically; however, the need remains for more effective spasticity management^7^. Moreover, given the high prevalence of spasticity after SCI, the use of preemptive therapy, which is administered before the development of spasticity, may be one of the options for controlling spastic symptoms. In this point of view, a novel intervention for the management of spasticity in the early phase of SCI is desirable.

It has been hypothesized that spasticity results from spontaneous activation of lower motor neuron pools located below the spinal lesions^8,9^. The hyperactivity of spinal motor neurons is attributable to changes in cell properties, such as reduction of potassium-chloride cotransporter (KCC2) expression^10^, and hypersensitivity of the serotonin receptors^11–13^. Serotonin (5-hydroxytryptamine; 5-HT) is one of the main neurotransmitters in the brainstem that activates spinal motor neurons^14^. The denervation of 5-HT fibers from the brainstem to the spinal cord causes 5-HT deficiency around lower motor neurons, which leads to the upregulation of the 5-HT_2A_ or the 5-HT_2C_ receptor in the neurons^15^. The increase of 5-HT receptors occurs during the course of experimental SCI and the upregulation of 5-HT receptors is correlated with the hyperexcitability of the spinal motor neurons^13,16–18^.

In this context, 5-HT_2_ receptor antagonists, such as cyproheptadine, have been utilized for the alleviation of spasticity^19,20^. In addition, the modification of the 5-HT system is also considered a therapeutic target in other central nervous diseases, such as depression. Because reduced 5-HT activity in the brain is known as one of the pathological mechanisms leading to depression^21^, antidepressants are aimed at restoring 5-HT levels as well as at the proper activation of 5-HT receptors^22^. Among these drugs, selective serotonin re-uptake inhibitors (SSRIs) are frequently prescribed; they are known to modify the 5-HT system by both elevating 5-HT concentration via blocking the 5-HT transporter (SERT) and decreasing the sensitivity of 5-HT receptors^23,24^. The use of SSRIs has been shown to exacerbate spasticity in human SCI patients^25,26^. Also, in our previous study, we reported that 2 weeks of SSRI administration from 4 weeks after contusive SCI exacerbated spastic symptoms as well as spinal hyperreflexia in a rodent SCI model^27^. It is considered that these results are due to the increase in the 5-HT concentration while 5-HT receptors were already upregulated. Therefore, we hypothesized that the upregulation of 5-HT receptors and spastic symptoms may be suppressed by an increase in the 5-HT concentration at the injury site via SSRI administration in the early stages of SCI. To demonstrate our hypothesis, in this study, we examined the effect of administration of an SSRI, escitalopram, starting from a day after the injury on spasticity after SCI. We found that four weeks of escitalopram administration in a rat model of contusive SCI could prevent the onset of spastic behaviors during the swimming test which examined from 3 weeks after the injury. Our study suggest that early SSRI administration may be used as a novel preemptive strategy for suppressing spasticity after SCI.

## Materials and methods

All methods were performed in accordance with the relevant guidelines and regulations.

### Animals, surgical procedures, and drug administration

The animals used in this experiment were 27 adult female Sprague–Dawley rats (200 to 300 g, Charles river Japan, Yokohama). The animals were anesthetized via intraperitoneal injection with a mixture of midazolam (2 mg/kg, Sandoz, Germany), butorphanol (2.5 mg/kg, Meiji Seika, Tokyo, Japan), and medetomidine (0.15 mg/kg, Kyoritsu Seiyaku, Tokyo, Japan). Prior to a surgical procedure, povidone-iodine was used for preoperative skin preparation and the body temperature of the anesthetized animals was kept by a heat pad. The rats received a 250-kilodyne (kd) contusive injury at the eighth thoracic vertebral level with an Infinite Horizon impactor device (Precision Systems and Instrumentation, VA, USA). After the surgery, we injected a single dose of an antibiotic (Baytril; 5 mg/kg, Bayer, Germany) to the rats. Each operated rat was housed separately in a 12:12 h light/dark cycle with controlled room temperature. Food and water were supplied ad libitum. We performed manual bladder expression twice daily until the injured animals could urinate independently.

All animal experiments were conducted in compliance with the ARRIVE guidelines under the jurisdiction of the Ministry of Education, Culture, Sports, Science and Technology, Japan. The experiments were approved by the animal ethics committee of the National Rehabilitation Center for Persons with Disabilities, Japan.

**Figure 1:**
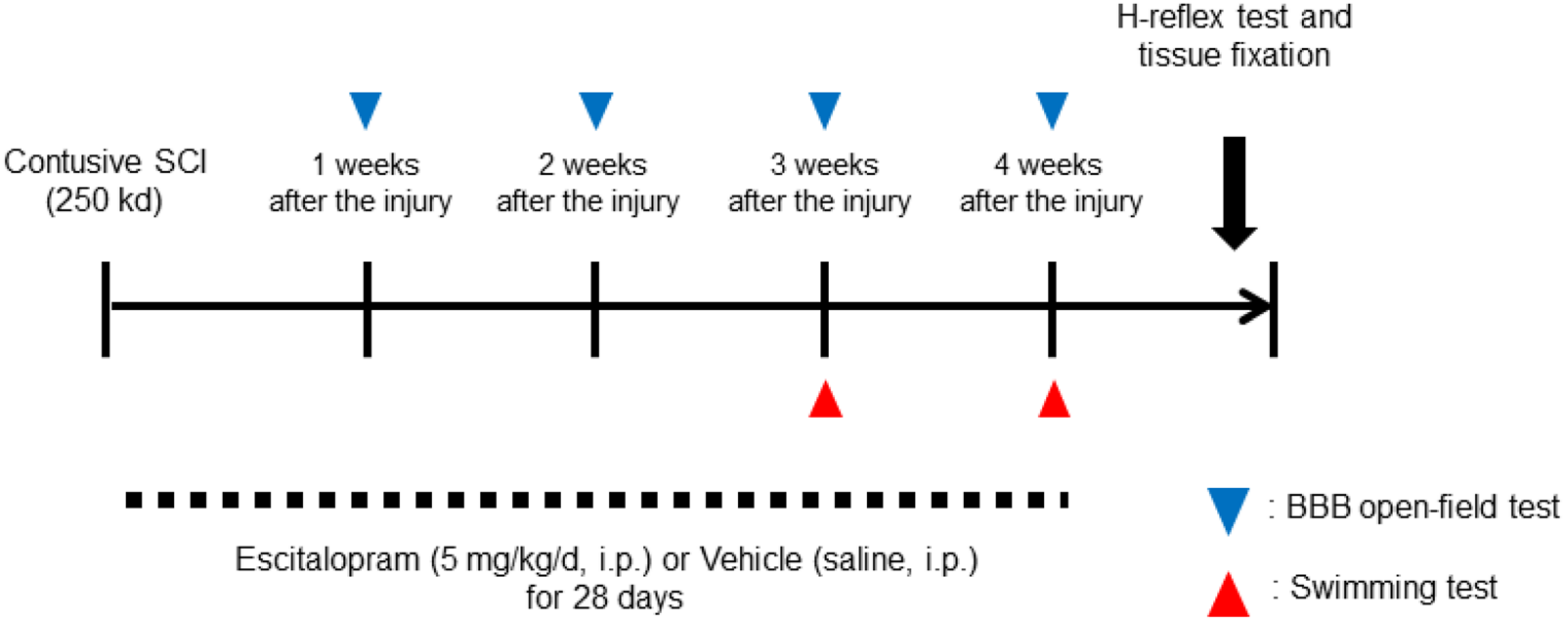
Experimental scheme in this study. Experimental timeline. Rats received a 250 kd contusive spinal cord injury. From day 1 after SCI, escitalopram or saline as vehicle were administrated once daily. The swimming test was performed at 3 and 4 weeks after the surgery. The H-reflex test and tissue fixation were performed 1 day after the final swimming test.

Escitalopram oxalate was purchased from Sigma (#E4786, MI, USA). After the surgery, 20 injured animals were assigned randomly into two groups: an escitalopram-treated and a vehicle-treated group (10 animals assigned to each group). The mean displacements of contusion were 1477.8 ± 49.1 μm for escitalopram-treated group and 1526.9 ± 60.8 μm for vehicle-treated group. Escitalopram (5 mg/kg, i.p.) was administered daily for 28 days from day 1 after SCI induction (Fig. 1). Drugs were injected once a day. Normal saline was used as a vehicle. On the day of the swimming and behavior tests, we injected the daily dose of escitalopram after the tests took place. Drug administration was not performed in a blinded manner. Seven rats were neither received SCI nor experimental interventions as for control.

### Swimming test and Basso, Beattie, and Bresnahan (BBB) locomotive scoring

**Figure 2:**
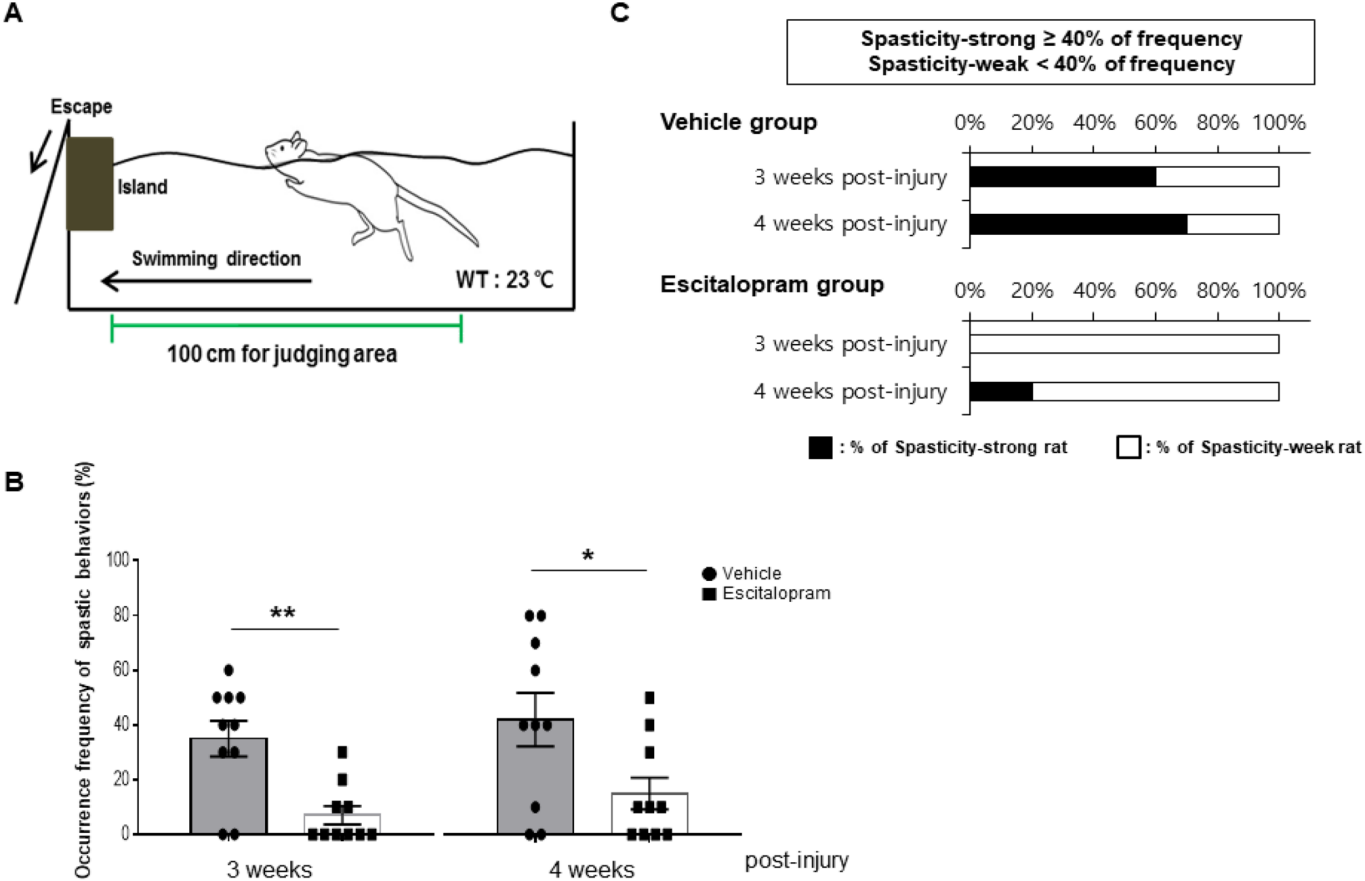
Effects of escitalopram on spastic behaviors during the swimming test and the ratio of spasticity-strong rats. (**A**) The configuration of the swimming test used in this study is presented. Spasm and clonus phases during the swimming test were counted in 10 swimming runs. *WT* water temperature. (**B**) The bar graph shows the mean occurrence frequency of spastic behavior (sum of spasm and clonus) during the swimming tests performed at 3 and 4 weeks after SCI (gray bar with round plots: vehicle-treated group, white bar with rectangular plots: escitalopram-treated group). *P < 0.05, **P < 0.01. (**C**) The ratios of spasticity-strong rats at 3 and 4 weeks after SCI. Injured rats were classified as “spasticity-strong” if they showed over 40% frequency of spasticity-positive swimming runs during the swimming test.

We performed the swimming test using a rectangular plexiglass chamber (150 × 14.5 × 40 cm) filled with tap water to a depth of approximately 20–23 cm (Fig. 2A). The details of this method were reported in our previous study^28^. Briefly, a total of 10 swimming runs were performed at 3 and 4 weeks after SCI induction (Fig. 1). We defined the occurrence of spastic behavior during the swimming test using the following criterion: if an SCI rat showed spastic behaviors, including the spastic or clonus phase, in a single swimming run, we counted the run as a ‘spasticity-positive’ run. Next, we calculated the percentage of spasticity-positive runs in the 10 swimming runs. The water temperature was maintained at 23 °C, which is considered optimal for swimming experiments using rats^29^. All the swimming tests were recorded with a HDR-CX700 camera (Sony, Japan) at 60 frames per s.

Regarding the BBB locomotor score^30^, we evaluated the hind limb locomotive function of injured animals in each group once per a week after SCI (Fig. 1). The rats were scored while moving freely on a table within 4-min assessment periods. All behavior tests were observed and analyzed by two skilled researchers. Investigators were not blinded to the experimental groups when scoring the behavioral tests.

### Hoffmann reflex (H-reflex) recording

To test the H-reflex, we chose the plantar muscle for its selectiveness and robustness of the H-reflex when the tibial nerve is stimulated in rodent SCI models^12,31–33^. Moreover, the changes in the plantar reflex were reported to be analogous to those of the other hind limb muscles, such as the gastrocnemius and anterior tibialis muscles, in a rat SCI model^33^.

We performed H-reflex test in rats according to our previously reported procedure^27^. Briefly, the day after the last escitalopram injection, the rats were anesthetized using chloral hydrate anesthesia (2.5 g/kg, i.p., Sigma). After anesthesia, the distal tibial nerve was exposed and a bipolar cuff was hooked to the nerve at the ankle level. A pair of recording electrodes was subcutaneously inserted into the plantar muscle of the hind limb on the same side. To evaluate the rate-dependent depression (RDD) of the H-reflex, we stimulated the nerve at 0.2, 0.5, 1, 2, and 5 Hz, respectively. The electromyography signals (sampling rate, 5000 Hz) were transferred to an amplifier (NEC Biotop 6R12, Nihon Kohden, Japan) and band-pass filtered (5–3000 Hz). We calculated the amplitudes of the M and H waves as an average peak-to-peak value of 15 waves of each waveform. The rate-dependent changes at each stimulation frequency were adjusted as a percentage of the response at 0.2 Hz. Data from the H-reflex tests were analyzed using the Spike2 software (Version 8, CED, Cambridge, England; http://ced.co.uk/).

### Immunohistochemistry

Animals were anesthetized with sodium pentobarbital and perfused with 4% paraformaldehyde (PFA) in PBS. Then, collected lumbar spinal cords, L4–L6 spinal cord level, were post-fixed additional 24 h with 4% PFA, cryo-protected by sequential incubation with 20% sucrose solution for 24 h, and embedded in OCT (Tissue-Tek, Japan). The spinal samples were sliced into 20 μm thick cryosections using a cryostat (Leica CM3050S, Leica Biosystems). The primary antibodies we used were as follows: anti-choline acetyltransferase antibody (ChAT, 1:100, AB144P, Merck Millipore, USA), anti-serotonin 2A receptor antibody (1:200, PC176, Merck Millipore, USA) and anti-KCC2 antibody (1:1000, ab49917, abcam, UK). The sections were incubated with secondary antibodies (Alexa Fluor 488 or 568 conjugated for each species matched type, 1:200, Thermo Fisher Scientific, USA) and DAPI (1:1000, Merck Millipore) for 2 h on the following day. The samples were observed using a BZ-9000 HS all-in-one fluorescence microscope (KEYENCE BZ-9000, Osaka, Japan).

### Data analysis

The area of immunoreactivity (IR) was calculated using the ImageJ software (Version 1.5, NIH, USA; https://imagej.nih.gov/). The 5-HT_2A_ receptor (5-HT_2A_R) levels were measured according to the methods previously described^28^. Briefly, we set the thresholds for each image, and measured the ChAT (choline acetyltransferase)-labeled areas in the soma and proximal dendrites of the spinal motor neurons located in the ventral horn of the lumbar spinal cord; the 5-HT_2A_R-labeled area corresponded to the ChAT-labeled area. The plasma membrane labeled KCC2 was measured according to previously reported methods by Boulenguez et al.^10^. Briefly, we adjusted the threshold using ImageJ and obtained KCC2 labeled pixels. Then, the pixels were divided at the somatic perimeter of ChAT-positive spinal motor neurons in the ventral horn of lumbar spinal cord. Five spinal tissue sections (each section was separated by over 300 μm) were used for 5-HT_2A_R and KCC2 microscopic image analyses. Investigators were not blinded to the experimental conditions when conducting the analyses.

### Statistical analysis

All the statistical analyses were performed using GraphPad Prism software (Version 7, GraphPad software, CA, USA). Statistically significant differences were determined by Student’s two-tailed *t*-test for the results of swimming test. The difference in RDD of the H-reflex was analyzed by one-way repeated-measures analysis of variance (ANOVA), and the histological quantification was analyzed by one-way ANOVA with post-hoc Bonferroni test for correction of multiple comparisons. The BBB scores were analyzed by Mann–Whitney U test. Results with *P* < 0.05 were considered to be statistically significant. The error bar represents the standard error of the mean (S.E.M).

## Results

### Escitalopram administration prevented spastic behaviors during the swimming test

SCI rats were treated with escitalopram or the vehicle from a day after the injury, when spastic symptoms had not yet manifested, and the effects of SSRI administration on spasticity were evaluated using the swimming test performed at 3 and 4 weeks after injury (Figs. 1, 2A). At each week, the rats swam 10 runs. We counted the number of runs accompanied by spastic behaviors and calculated the occurrence frequency of spastic behaviors (i.e., we quantified 40% of the frequency of spastic behaviors during the swimming test, if a SCI rat showed four counts of spasticity-positive runs among 10 swimming runs).

The vehicle-treated group showed average 13.0% ± 3.7% of counted clonus phase and average 22.0% ± 5.3% of spasm phase in 10 runs at 3 weeks after SCI and 13.0% ± 4.2% and 29.0% ± 8.4%, respectively, at 4 weeks after SCI. On the contrary, the escitalopram-treated group presented average counted clonus phase and spasm phase in 10 runs as 3.0% ± 2.1% and 4.0% ± 2.2%, respectively, at 3 weeks after SCI and 3.0% ± 2.1% and 12.0% ± 5.3%, respectively, at 4 weeks after SCI In total, the escitalopram-treated group presented significantly reduced spastic behaviors compared to the vehicle-treated group during the swimming test at both 3 and 4 weeks post-injury (vehicle-treated group: 35.0% ± 6.5%, escitalopram-treated group: 7.0% ± 3.4% at 3 weeks post-injury; vehicle-treated group: 42.0% ± 9.8%, escitalopram-treated group: 15.0% ± 5.8% at 4 weeks post-injury; n = 10 per group, *t*_18_ = 3.810, *P* = 0.0013 for 3 weeks post-injury and *t*_18_ = 2.377, *P* = 0.0287 for 4 weeks post-injury, Student’s two-tailed *t*-test; Fig. 2B).

It has been recognized that even if the same severity of contusive injury was applied, the injured rats presented various levels of severity of spasticity^28,29^. Therefore, we grouped them into two groups, namely spasticity-strong and spasticity-weak groups, based on their score on the swimming test with threshold at 40% frequency of spastic behaviors, including both clonus and spasm phase, during the swimming test^28^. According to this criterion, the ratios of spasticity-strong rats in the vehicle-treated group were 60% at 3 weeks and 70% at 4 weeks after SCI. In contrast, the ratios of spasticity-strong rats in the escitalopram-treated group were 0% and 20% at 3 and 4 weeks post SCI, respectively (Fig. 2C). These results suggest that 4 weeks of SSRI administration is effective in suppressing the development of spasticity after traumatic SCI.

### Rate-dependent depression of the H-reflex was unchanged

Next, we tested the rate-dependent depression (RDD) of the Hoffman reflex (H-reflex) in order to examine spinal motor neuronal excitability of SCI animals. The H-reflex is frequently used as a classic neurological tool for determining spasticity in both human patients and animal models^31,34,35^. Briefly, the M-wave which is an output of direct stimulation of motor neuronal axons does not altered upon the RDD of the H-reflex test. On the contrary, in the normal condition, the H-wave is changed by different stimulation rates, which reflects a monosynaptic reflex via the afferent Ia sensory fibers and the spinal motor neurons.

**Figure 3:**
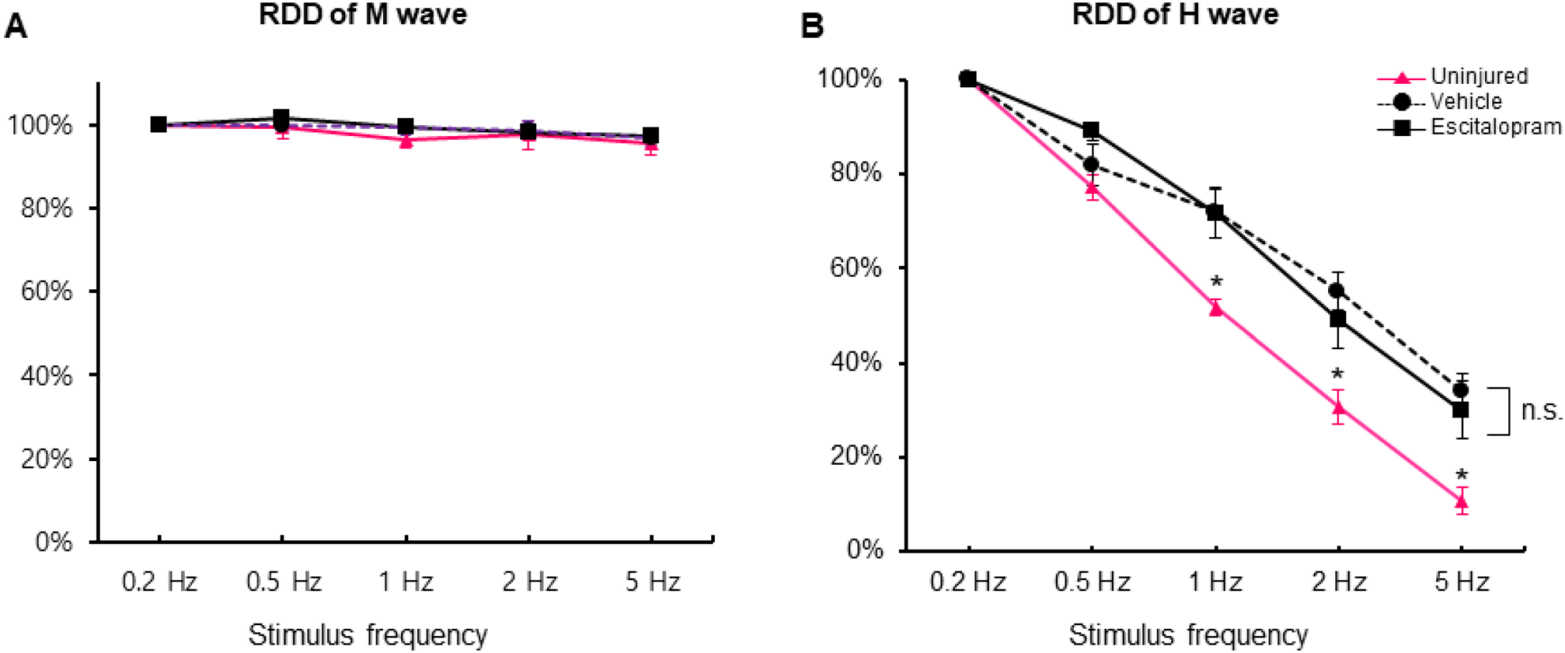
Rate-dependent depression (RDD) of the H-reflex in the experimental groups. (**A**) The rate-dependent depression (RDD) of the M wave in each group is shown with different stimulation rates. (**B**) Graphs present the RDD of the H-wave in each group (magenta line with triangular plots: uninjured group, dashed line with round plots: vehicle-treated group, black line with rectangular plots: escitalopram-treated group). *P < 0.05, *n.s.* non-significant.

In comparison between escitalopram-treated SCI rats and vehicle-treated SCI rats, the M-wave remained unchanged after the stimulation frequency was applied (Fig. 3A). We observed that RDD of the H-wave of both the escitalopram and vehicle-treated groups significantly decreased compared to that of uninjured rats at 1, 2, and 5 Hz stimulation rates (n = 7 for uninjured, n = 10 for escitalopram-treated and vehicle-treated groups, *F*_2,25_ = 4.912, *P* = 0.016 for 1 Hz, *F*_2,25_ = 5.171, *P* = 0.013 for 2 Hz, *F*_2,25_ = 5.341, *P* = 0.012 for 5 Hz stimulation, one-way repeated-measures ANOVA with post-hoc Bonferroni tests; Fig. 3B). However, we did not find any differences between the escitalopram-treated and vehicle-treated groups (*P* > 0.05, Post-hoc Bonferroni comparisons; Fig. 3B).

### The expression of 5-HT_2A_ receptor decreased after 4 weeks of SSRI administration whereas KCC2 was not altered

**Figure 4:**
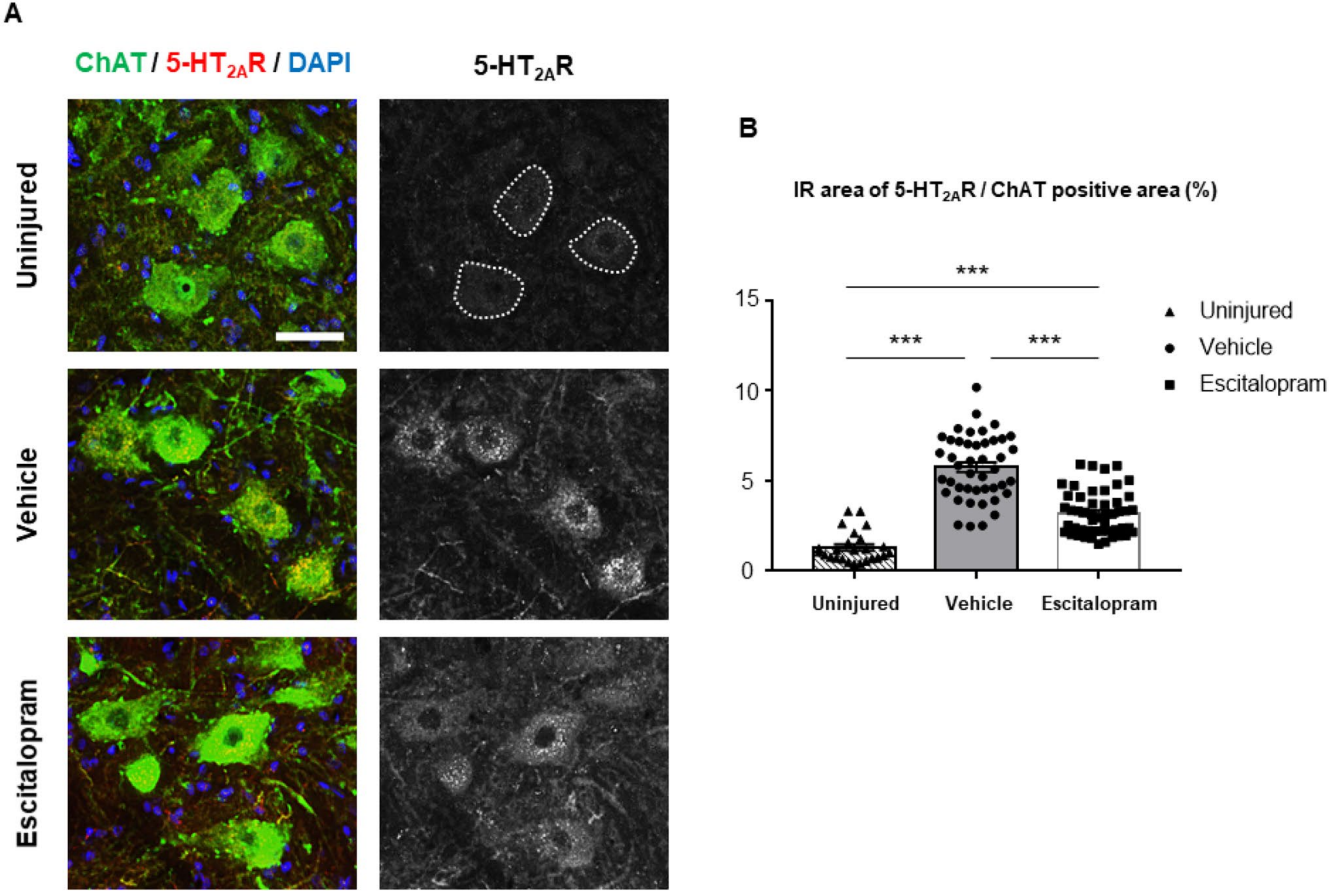
Change of immunoreactivity of the 5-HT_2A_ receptor in the lumbar spinal cord motor neurons. (**A**) Representative microscopic images of immunoreactivity (IR) of the 5-HT_2A_ receptor (5-HT_2A_R; red) on the lumbar motor neurons of each group are shown. As shown at the top, spinal motor neurons were stained by the anti-ChAT (green) antibody and the IR area of the 5-HT_2A_R was confined to the ChAT-stained area (white lines in the binarized image indicate ChAT margins). The nuclei have been stained by 4′,6-diamidino-2-phenylindole (blue). Scale bar: 50 μm. (**B**) The bar graph with plots presents the quantitative analysis of the IR area of the 5-HT_2A_R in the ChAT-positive area of lumbar motor neurons of each group (dashed bar with triangular plots: uninjured group, grey bar with round plots: vehicle-treated group, white bar with rectangular: escitalopram-treated group). ***P < 0.001.

We further explored the histological changes of the lumbar spinal cord after 4 weeks of escitalopram administration. For this purpose, we performed immunostaining and analyzed the expression of the 5-HT_2A_ receptor and KCC2, which are considered to have an important role in the onset of spasticity and its severity. First, consistent with our previous report^28^, the immunoreactivity (IR) of 5-HT_2A_R in the lumbar spinal motor neurons 4 weeks after SCI was significantly higher than that in uninjured animals (n = 5 for uninjured, n = 10 for the escitalopram-treated group, n = 9 for the vehicle-treated group, 4–5 tissue sections were analyzed per each subject, *F*_2,116_ = 87.12, *P* < 0.001, one-way ANOVA with post-hoc Bonferroni tests; Fig. 4A,B). After the SSRI intervention, the IR area of the 5-HT_2A_R in the lumbar motor neurons of escitalopram-treated group was significantly lower compare to the vehicle-treated group (*P* < 0.001, Post-hoc Bonferroni comparison; Fig. 4B).

**Figure 5:**
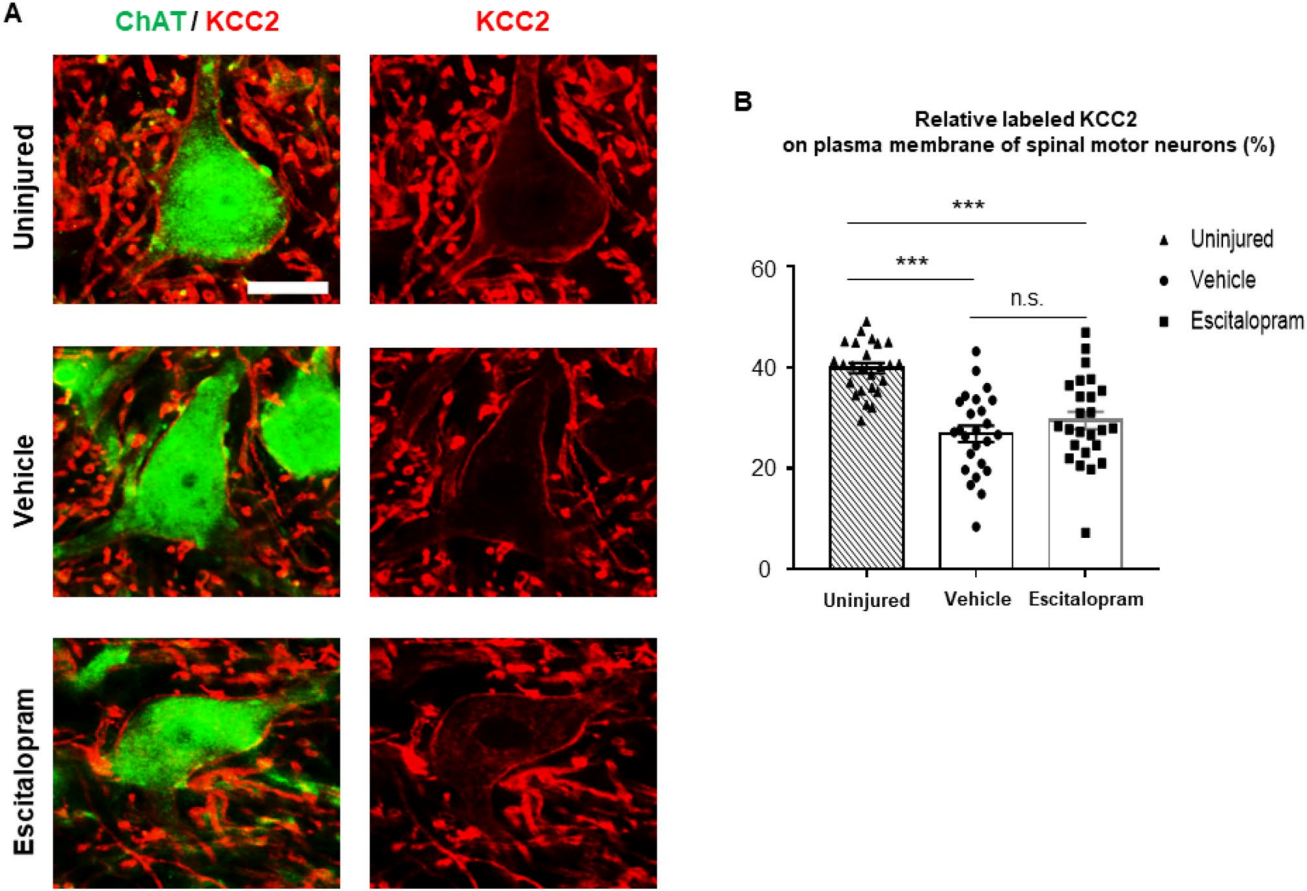
Plasma membrane labeled KCC2 immunostaining on the lumber spinal motor neurons. (**A**) Representative microscopic images of anti-KCC2 immunostaining (red) on the ChAT positive-lumbar motor neurons (green) of each group are presented. Scale bar: 25 μm. (**B**) Quantitative analysis of plasma membrane labeled KCC2 of each group, normalized to uninjured and shown as a bar graph (dashed bar with triangular plots: uninjured group, grey bar with round plots: vehicle-treated group, white bar with rectangular: escitalopram-treated group). ***P < 0.001, *n.s.* non-significant.

Next, we examined the expression of KCC2 on the spinal motor neurons. We observed a significantly reduced membrane labeled KCC2 signal on the spinal motor neurons 4 weeks post-SCI, in line with previous reports^10,36^ (n = 5 for each group, total 150 motor neurons, 5 tissue sections were analyzed per each subject, *F*_2,72_ = 21.36, *P* < 0.001, one-way ANOVA with post-hoc Bonferroni tests; Fig. 5A,B). Despite of 4 weeks of escitalopram administration, we did not find a significant increase of membrane KCC2 expression on the lumbar spinal motor neurons compared to the vehicle-treated group (*P* > 0.5, Post-hoc Bonferroni comparison; Fig. 5B).

### Systemic escitalopram administration did not improve open-field locomotor behaviors after SCI

**Figure 6:**
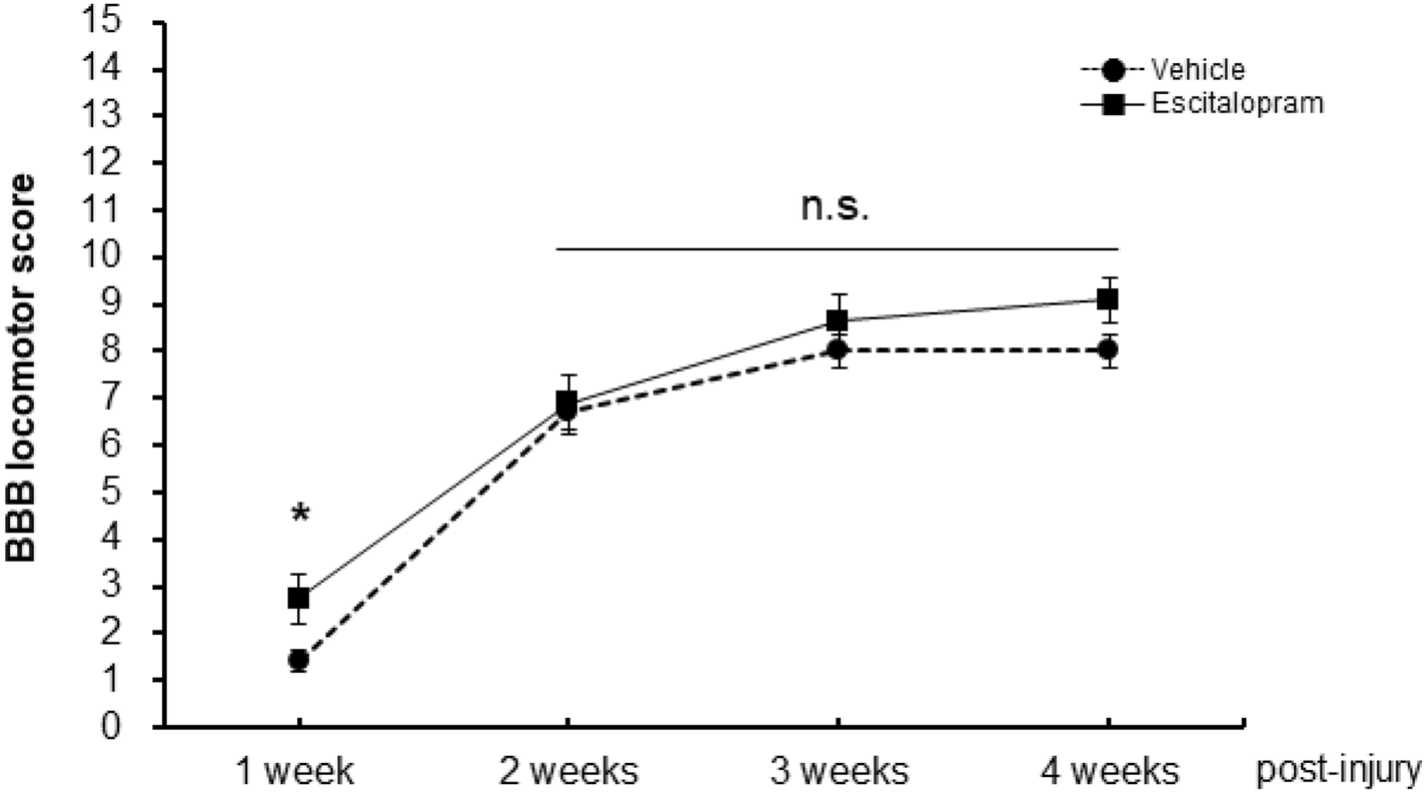
Evaluation of hind limb locomotor scoring. The graph shows the weekly Basso, Beattie, and Bresnahan locomotor score of each group (dashed line with round plots: vehicle-treated group, black line with rectangular plots: escitalopram-treated group). *P < 0.05, *n.s.* non-significant.

In the course of escitalopram administration, the escitalopram-treated group showed a significantly increased BBB locomotor score compared to the vehicle-treated group at 1 week post SCI (n = 10 per group, *P* = 0.0316, Mann–Whitney U test; Fig. 6). However, in the later time points, there were no statistical differences in BBB locomotor scores between the two groups (*P* > 0.05, Mann–Whitney U test; average BBB score of the vehicle-treated group: 8.0 ± 0.3, average BBB score of the escitalopram-treated group: 9.1 ± 0.5 at 4 weeks post-SCI).

## Discussion

In the present study, we examined the effect of early administration of escitalopram for 4 weeks post-injury on spasticity using a contusive SCI rat model. Our results indicate that the SSRI treatment suppressed spastic behaviors after SCI and decreased the ratio of spasticity-strong rats compared to the effects of the vehicle treatment. Moreover, we observed suppression of the 5-HT_2A_R upregulation on the spinal motor neurons below the injury site. However, we did not find significant alterations in the spinal monosynaptic reflex nor changes in KCC2 expression in the spinal motor neurons with escitalopram administration.

The swimming test has been used for both partial transection and contusive SCI rat models to evaluate the severity of spasticity^28,29^. Previously, we confirmed that this test enables to discriminate spasticity-strong and -weak rats after the injury by quantifying occurrence frequency of spastic behavior during the test. As is observed in the clinical time course, spastic behavior during swimming test increases in the subacute phase, 3–4 weeks after injury in rodent models^5–7,29^. At 4 weeks after injury in the present study, 70% vehicle-treated rats, as opposed to only 20% escitalopram-treated rats, manifested the spasticity-strong phenotype. Although we did not examine spasticity beyond 4 weeks after SCI, it is noteworthy that escitalopram treatment prevented spastic behaviors during the development period of spasticity. This indicates that early SSRI administration can control the onset of spastic symptoms.

When SCI animals were treated with an SSRI from 4 weeks after SCI, spastic behaviors were exacerbated^27^. Furthermore, clinical studies reported that human SCI patients with spasticity showed worsened symptoms after SSRI administration^25,26^. Therefore, once the spasticity has become symptomatic, it is considered that an increase in the 5-HT concentration exacerbates spastic symptoms. However, as shown in the present study, SSRI administration before the development of spasticity could suppress the onset of spastic behaviors. Although we did not examine spasticity beyond 4 weeks after SCI, it is noteworthy that escitalopram treatment prevented spastic behaviors during the development period of spasticity. This indicates that early SSRI administration can control the onset of spastic symptoms.

The gradual development of spasticity after SCI is considered to be correlated to the changes of molecular property in spinal motor neurons. Hypersensitivity of the 5-HT receptor after SCI is one of the crucial factors in spasticity and leads to neuronal hyperactivity^11,17,37^. Significant upregulation of 5-HT_2A_R and 5-HT_2C_R was reported after transection SCI. Although 45 days are required before the expression of 5-HT_2C_R increases after SCI, 5-HT_2A_R expression is rapidly upregulated from the day after the injury^16,18^. Moreover, recent studies have demonstrated that mRNA expression of 5-HT_2C_R does not significantly change after SCI in rodent models while the expression of 5-HT_2A_R is significantly upregulated^36,38^. Previously, we reported that the upregulated 5-HT_2A_R expression on the lumbar spinal motor neurons is correlated to severer spastic behaviors during the swimming test^28^. Thus, we consider that escitalopram treatment suppressed spastic symptoms through regulation of the 5-HT_2A_R expression.

The ability of anti-depressants, including SSRIs, to downregulate 5-HT receptors has been documented in human and animal studies^22,39,40^. However, the specific mechanisms have not been fully elucidated. In the current SCI model, the increased level of 5-HT concentration by SSRI which blocks serotonin transporter may have compensated 5-HT deficiency due to 5-HT fiber denervation, thus, ameliorated the upregulated 5-HT receptor expression. Furthermore, the non-selective affinity of SSRIs to 5-HT receptors may also cause desensitization of the 5-HT receptors^41^. In this study, we neither confirmed 5-HT concentration in the spinal cord after the 4 weeks of intervention nor determined the non-selective binding of escitalopram to the 5-HT_2A_ receptor. Further studies for the detail mechanisms of reduction in 5-HT receptors may provide the information about which SSRIs are optimal to reduce spasticity after SCI.

We anticipated that suppressed spastic behaviors in escitalopram-treated rats would be accompanied by recovery of the reflex in the spinal circuit. However, we did not observe differences in RDD of the H-reflex between the escitalopram-treated and vehicle-treated groups. Additionally, we did not observe significant differences in KCC2 expression in the spinal motor neurons below the injury site between the two groups. KCC2 facilitates inhibitory inputs of GABA and glycine receptors to neuronal cells by maintaining low intracellular Cl^−^ concentration^42^. After SCI, down-regulation of KCC2 occurs and leads to the increase in intracellular Cl^−^ concentration, which reduces inhibitory GABAergic inputs^10^. The correlation between the expression level of KCC2 and H-reflex has been reported by Cote et al., who reported that bicycling exercise recovered KCC2 expression in the spinal motor neurons and improved RDD of the H-reflex in a complete spinal transection rat model^43^. Therefore, we assume that the escitalopram treatment did not alter the expression of KCC2, which resulted in the similar RDD of the H-reflex between the two groups. The discrepancy between suppressed spastic behaviors in the swimming test and unchanged H-reflex after escitalopram treatment may be due to the complexity of pathological conditions underling spasticity; it includes the supraspinal descending pathway (5-HT system) and the monosynaptic Ia afferent reflex pathway, among others^7^. Regarding this point, we assume that the swimming test may well reflect the condition of the supraspinal descending pathway, while the H-reflex test under anesthesia may reflect the excitability of motor neurons against peripheral afferent inputs.

As for the locomotive ability, the hindlimb function of escitalopram-treated group was not superior to that of vehicle-treated group. Since the severity of our SCI model is around score 8 of BBB score, which indicates injured rats cannot take weight bearing posture, changes in spasticity cannot be reflected in the BBB score at this severity. In addition, escitalopram-induced changes in 5-HT concentration around spinal motor neurons may not be sufficient to augment neural excitation and muscle contraction compared to injections of serotonin or 5-HT agonist, which are reported to enhance locomotor functions and coordination after SCI^14,44,45^.

Based on the findings of this study, we suggest that early SSRI administration may be useful for controlling the development of spasticity after traumatic SCI as a preemptive therapy. SSRIs are a commonly prescribed drug for psychological cares and their effectiveness and safety have been proven. Considering that many patients with SCI exhibit depression after the injury, especially in the acute stage^46^, we speculate that early SSRI administration will be helpful in dealing with depression as well as the onset of spasticity after SCI.
